# Observe Before You Leap: Why Observation Provides Critical Insights for Formative Research and Intervention Design That You'll Never Get From Focus Groups, Interviews, or KAP Surveys

**DOI:** 10.9745/GHSP-D-17-00328

**Published:** 2018-06-27

**Authors:** Steven A. Harvey

**Affiliations:** a Social and Behavioral Interventions Program, Department of International Health, Johns Hopkins Bloomberg School of Public Health, Baltimore, MD, USA.

## Abstract

Formative research is essential to designing both study instruments and interventions in global health. While formative research may employ many qualitative methods, focus group discussions and in-depth interviews are the most common. Observation is less common but can generate insights unlikely to emerge from any other method. This article presents 4 case studies in which observation revealed critical insights: corralling domestic poultry to reduce childhood diarrhea, promoting insecticide-treated bed nets (ITNs) to prevent malaria, evaluating skilled birth attendant competency to manage life-threatening obstetric and neonatal complications, and assessing community health worker (CHW) ability to use malaria rapid diagnostic tests (RDTs). Observation of Zambian CHWs to design malaria RDT training materials revealed a need for training on how to take finger-stick blood samples, a procedure second nature to many health workers but one that few CHWs had ever performed. In Lima, Peru, study participants reported keeping their birds corralled “all the time,” but observers frequently found them loose, a difference potentially explained by an alternative interpretation of the phrase “all the time” to mean “all the time (except at some specific seemingly obvious times).” In the Peruvian Amazon, observation revealed a potential limitation of bed net efficacy due to the built environment: In houses constructed on stilts, many people sleep directly on the floor, allowing mosquitoes to bite from below through gaps in the floorboards. Observation forms and checklists from each case study are included as supplemental files; these may serve as models for designing new observation guides. The case studies illustrate the value of observation to clearly understanding clinical practices and skills, details about how people carry out certain tasks, routine behaviors people would most likely not think to describe in an interview, and environmental barriers that must be overcome if an intervention is to succeed. Observation provides a way to triangulate for social desirability bias and to measure details that interview or focus group participants are unlikely to recognize, remember, or be able to describe with precision.

## Abstract (precis)

Four case studies show how observation can uncover issues critical to making a health intervention succeed or, sometimes, reveal reasons why it is likely to fail. Observation can be particularly valuable for interventions that depend on mechanical or clinical skills; service delivery processes; effects of the built environment; and habitual tasks that practitioners find difficult to articulate.

## INTRODUCTION

Let's play a quick game of word association: If I say “formative research,” what's the first word or phrase that comes to mind? Some of you, thinking of purpose, might say that formative research is what you do before designing a behavior change campaign. Others, thinking of methods, might say “focus groups.” Both would be wrong. Well, at least partially wrong.

Formative research *is* important to the design of behavior change campaigns, but it serves many other purposes as well. It is essential to developing research instruments and global health interventions of many kinds.^1^^–^^4^ It can provide the basis for assessing clinical practice, determining how to measure intervention outcomes, planning quality improvement initiatives, and understanding many other aspects of global health programming.^5^^–^^14^ As medical anthropologist Margaret Bentley explains^15^:

*The purpose [of formative research] is to provide input into the design of a research study or intervention, including the identification of target populations and appropriate recruitment, retention or consent strategies, development of assessment or evaluation measures, and refinement of intervention components. Formative research allows community participation in the design of research and program protocols, which leads to greater community acceptance.*

So formative research is about much more than just behavior change interventions.

Now, what about methods? If you want to *do* formative research, how should you go about it? Formative research can incorporate many methods, both qualitative and quantitative. Focus groups tend to be the most common, perhaps because they are most familiar. Interviews and knowledge, attitude, and practice (KAP) surveys are also popular. However, as you've probably gathered by now, I'm going to argue that those methods are often insufficient. If you're doing formative research, you should also consider observation.

If you're doing formative research, you should consider observation.

Researchers seem more hesitant about observation than other methods, perhaps because they don't know how to do it, consider it too labor-intensive or costly, feel uncomfortable with the idea of watching other people, or worry about reactivity—the phenomenon where those being observed change their behavior due to the observer's presence.^16^^–^^18^ But observation can generate insights you won't get using any other method. And those insights can often prove critical.

In this article, I present 4 case studies on different global health topics, from corralling domestic poultry to measuring the competency of skilled birth attendants (SBAs).^19^^–^^21^ These examples illustrate some of the scenarios in which observation—both structured and unstructured—can be useful, and they highlight the types of insights it can provide. In each case study, observation yielded critical information that would have been difficult or impossible to obtain any other way. For each case study, I provide a brief description of the research and the context from which it was drawn, then focus more extensively on the observational methods used and the unique insights they generated. Complete descriptions of the original research can be found elsewhere.^22^^–^^28^ I've provided the observation instruments used for each case study as supplemental files.

### Ethics Review

The research cited in case studies #1 and #2 was reviewed and approved by the Institutional Review Board of the Johns Hopkins Bloomberg School of Public Health in Baltimore, MD, USA, and by the Ethics Committee of the Asociación Benéfica PRISMA in Lima, Peru. The research cited in case study #3 was reviewed for compliance with the ethics guidelines of the Quality Assurance Project funded by the United States Agency for International Development and approved by Ministry of Health ethics committees or their equivalent in each study country. The research cited in case study #4 received ethics approval from the World Health Organization Special Programme for Research and Training in Tropical Diseases (WHO/TDR) and by the Tropical Disease Research Centre Ethics Committee – Ndola, Zambia.

## CASE STUDY #1: CORRALLING DOMESTIC POULTRY TO REDUCE CHILDHOOD DIARRHEA IN LIMA, PERU

### Background

*Campylobacter jejuni* is a common bacterial contributor to diarrheal disease worldwide.^23^^,^^29^^–^^31^ The bacteria is found almost universally in the intestinal tracts of chickens and can be transmitted to humans from contact with chicken feces or consumption of undercooked chicken.^23^^,^^32^^–^^36^ In the shanty town outside Lima, Peru, where this study took place, the link between *C. jejuni* in domestic poultry and childhood diarrhea has been established for decades and confirmed repeatedly.^23^^,^^32^^,^^37^

### Study Context and Observation Methods

The observations described here took place as formative research for a trial to test whether corralling free-range chickens and other domestic poultry would reduce *Campylobacter-*associated diarrhea by minimizing contact between children and birds.^23^ The research team recruited 12 local families raising domestic poultry, built corrals for the poultry at each household, and asked each family to test the corral for 8 weeks. A study team member made weekly visits to each household to complete a 19-item structured observation form (Supplement 1) with space to record variables such as number of birds present; number inside and outside each corral; visual evidence that birds might have been outside the corral recently (e.g., feathers or bird droppings in the yard or inside the house); interaction, if any, between birds and children; cleanliness and structural soundness of each corral; and presence and cleanliness of food and water. The weekly visits were carried out at preselected random times during daylight hours Monday–Saturday. Participants were not notified of visits in advance. This unannounced random schedule made it possible to observe the natural state of each household and corral on different days of the week and at different times of day. In addition, the project sociologist made 3–4 random semi-structured spot checks per household over the 8-week period (30 total across the 12 participating households) noting whether, at the moment of arrival, birds were corralled, children were interacting with birds, birds had adequate food and water, and corrals were in good condition. The sociologist took unstructured notes on anything he judged relevant to feasibility or acceptability of corralling.

### Critical Findings

#### Extent of Corralling

In interviews, participants stated that they kept their birds corralled “all the time.” However, observers found birds loose during 13% of observation visits and 33% of spot checks. Asked about this difference, participants clarified that they let the birds out at certain times such as while cleaning the corrals or to give them time to play (*recrearse*)—an activity owners considered essential to their birds' well-being.

Why did participants say they kept their birds corralled all the time when they really didn't? One possible reason is courtesy bias: The project had built them corrals, and so participants may have felt they would disappoint us or seem ungrateful by admitting they didn't always use them. Another possible reason is that they meant something different than we did by “all the time.” Participants took for granted that—like themselves—everyone would understand the need to let birds loose at certain times for practical or health reasons, a “fact” seemingly so obvious as to be unworthy of mention. “All the time” really meant “all the time except at certain (presumably obvious) specific times.” Had we relied solely on interviews (reported behavior), we might never have known that birds were sometimes loose or might never have thought to ask why. Triangulation between what people told us and what we observed revealed critical information about why the intervention might not work.

Participants took for granted that—like themselves—everyone would understand the need to let birds loose at certain times for practical or health reasons, a “fact” seemingly so obvious as to be unworthy of mention.

#### Sufficient Food and Water

**FIGURE 1:**
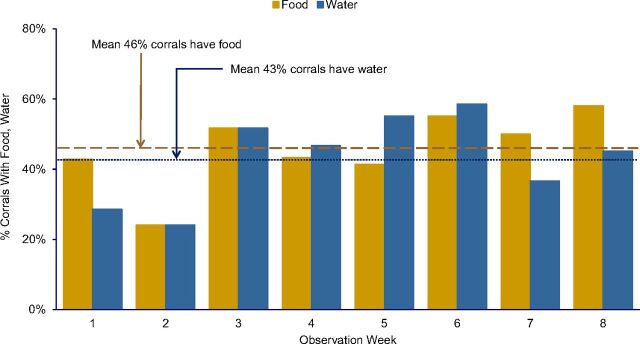
Percentage of Domestic Poultry Corrals Containing Food or Water During Weekly Random Observations, Lima, Peru (N=122 Observations)

For the local population, one advantage of raising loose poultry was that the birds could find their own food and water. With a corral, the household needed to provide a constant supply of food and water and maintain hygienic conditions. As shown in Figure 1, both structured observations and spot checks revealed that over the 8-week surveillance only 46% of corrals had food and only 43% had water. Further, corral floors were often wet after birds overturned their water dishes, and food was often rotting. In earlier interviews, participants had expressed concern that corralling would be unhealthy for their birds. Observations made clear that a corralling intervention might validate these concerns unless participants received training on how to keep corrals clean and corralled birds healthy. The data also showed that corralling took more time and effort since someone had to clean the corrals regularly and ensure availability of food and water.

#### Contact Between Poultry and Children

The primary objective of corralling was to break the *Campylobacter* transmission cycle by separating birds from children. Observations demonstrated that children took a keen interest in the new corrals, often swinging on the doors, sticking their fingers through the mesh, or entering to play with the birds. Attempts to childproof corrals with latches or convince parents to keep children away were largely ineffective: Observers continued to encounter children inside. Parents explained that this was natural and appropriate: They wanted their children to grow up around animals. Children as young as 3 were assigned to collect eggs every day. Instead of isolating children from *C. jejuni*, observations suggested that corralling actually concentrated exposure. This may help explain the finding from a later study that rates of *Campylobacter*-associated diarrhea among children under 6 were 2 to 7 times *higher* in corralling households than non-corralling households with the same number of chickens.^38^ Without observation, we might have missed the child-bird contact.

#### Handling of Poultry Manure

One contributor to child *Campylobacter* exposure not revealed in interviews was household handling of chicken manure. With manure now concentrated in a smaller space, poultry-raising households began to collect it to use as fertilizer. Observers documented that manure removed from coops was often stored in tin cans or buckets outside the coop within easy reach of children. Uncovered storage also allowed the wind to scatter dried manure around the outside of the living area, thus increasing potential contact.

#### Contrast Between Human and Bird Habitation

**FIGURE 2:**
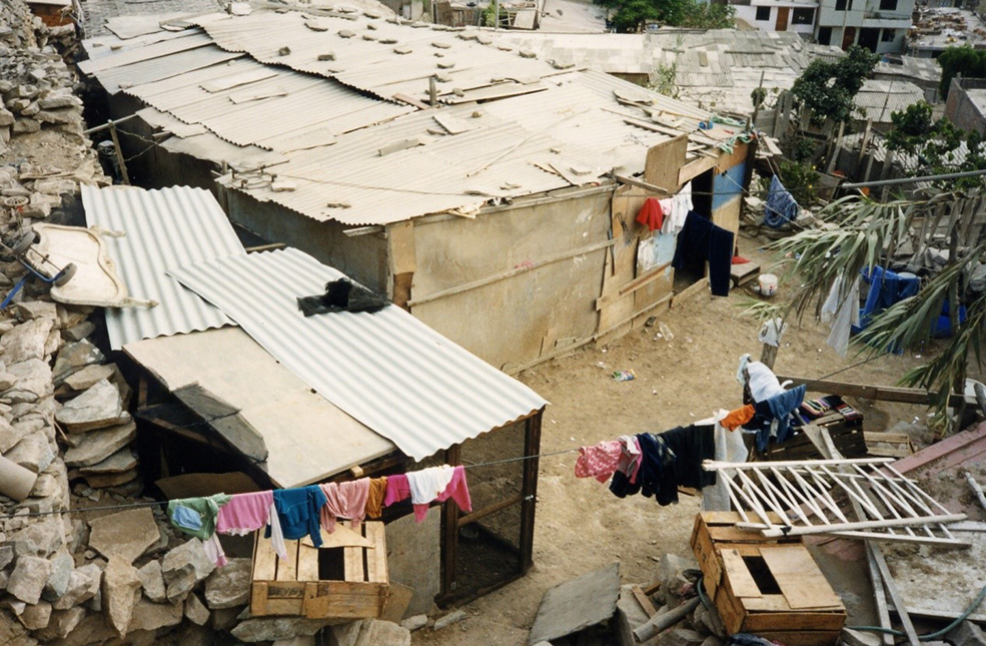
Contrast Between Human and Animal Living Spaces Documented Through Observation, Las Pampas de San Juan de Miraflores, Peru Project-constructed poultry corral (left foreground) vs. human habitation (center background). Project participants sometimes joked that the birds in the project enjoyed a better standard of living than the people. © 1999 Steven Harvey.

Though not part of formal data collection, observers also noted the contrast between human and animal living space. Residents of this area had settled outside Lima as squatters, often after fleeing rural terrorism in the 1980s. Most worked as casual laborers, domestic servants, or textile piece-workers earning the equivalent of $4.00 to $5.00 per day. Many lived in houses cobbled together from discarded materials, often scavenged from construction sites or garbage dumps. Corrals, though built as cheaply as possible, were made from new material at an average cost of $60.00 per household. Figure 2 shows a project-constructed corral to the left with the human habitation in the center. After receiving their corrals, more than 1 participant joked that their birds now enjoyed a higher standard of living than the human members of the family. Documenting this contrast offered a perspective beyond that likely to be achieved through interviews or focus groups alone.

## CASE STUDY #2: BED NETS FOR MALARIA PREVENTION IN THE PERUVIAN AMAZON

### Background

Malaria was virtually eliminated from the Peruvian Amazon during the 1970s and 1980s but began to reappear sporadically in the mid-1990s, culminating in an epidemic outbreak in 1997.^39^ In response that year, the Peruvian Ministry of Health began distributing ITNs to affected communities. This case study involves observations carried out to evaluate the social acceptability of ITNs and to assess their potential efficacy based on human behavior during the peak biting hours of local malaria-transmitting mosquitoes.

### Study Context and Observation Methods

The study took place in 1 peri-urban community and 3 rural villages, all within 30 km of Iquitos, the Peruvian Amazon's largest city. Over 9 months, 4 observers carried out 1 dusk-to-dawn observation in each of 60 households. Upon arrival, the observer used a structured form (Supplement 2) to collect information about the number, ages, and relationships of household occupants; the number and types of sleeping spaces; and the number and types of bed nets. The observer then took unstructured notes at 5-minute intervals throughout the night, recording the location and activities of each household member. Most households consisted of a wooden platform on stilts raised about 2 meters off the ground and covered with a thatched roof. These structures had few rooms or interior dividers, so observers could follow most household activities from a single vantage point.^17^^,^^40^

### Critical Findings

#### Net Use During Peak Biting Hours

**FIGURE 3:**
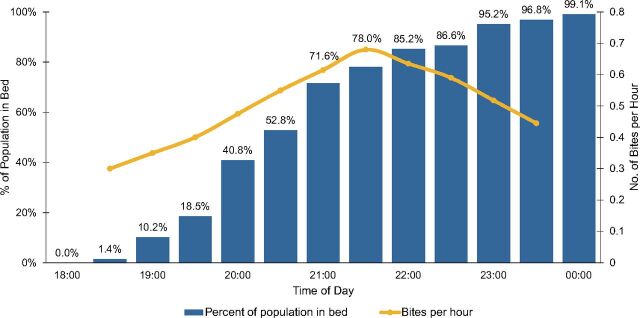
Percentage of the Population in Bed by Half-Hour (N=60 Observations) Compared With *Anopheles darlingi* Feeding Behavior,^a^ Department of Loreto, Peru ^a^ Data on mosquito feeding behavior come from Vittor (2003).^41^

A key concern about ITN effectiveness in the Americas is whether people are likely to be inside a net during the hours when local malaria-transmitting mosquitoes bite. Observation allowed us to systematically document net use. As shown in Figure 3, people began to enter their nets for the night as early as 7:00 p.m., but only about half the population was inside a net by 8:30 p.m. and slightly less than 80% by 9:30 p.m., the peak biting hour for *Anopheles darlingi*, the Amazon's most important malaria vector.^42^ This suggests that ITNs might be somewhat effective, but not as effective as in Africa where principal vector species feed later at night. Rather than observing all night, we might have simply asked people what time each member of the household went to bed the previous night, but in a setting where few people had watches or clocks, it would have been hard for them to respond with much precision. Social desirability bias might also have affected people's reports about their own behavior: At the time, the Ministry of Health was running a campaign encouraging people to enter their nets at dusk—a practice unlikely to be feasible in an area near the equator where the sun sets around 6:30 p.m. throughout the year.

#### Multiple Entries and Exits

One unanticipated finding was the number of times people enter and exit ITNs during the night.^43^ Each time the net is lifted, mosquitoes have an opportunity to enter. Parents who share nets with children may spend considerable time outside the net unprotected after their children have gone to sleep. The Table shows an example of a single sleeping space occupied by a 23-year-old mother and her 2-year-old son. The net was lifted a total of 20 times between 7:00 p.m. and 6:30 a.m. The mother spent 195 minutes outside the net between the first time she entered with her son at 7:00 p.m. and the time both of them got out of bed at 6:30 the next morning.

**TABLE.** Observational Bed Net Entry and Exit Data From a Single Sleeping Space With 2 Occupants, a 23-Year-Old Mother and Her 2-Year-Old Son, Peruvian Amazon

Time	Entry (+)/Exit (−) [Minutes Outside Net Before Reentering]		Comments
	Mother (P1)	Child (P2)	
19:00	+/− [145 min]	+	P1 gets into net with P2, then gets back out, does housework, cares for pigs, chickens.
20:50			P1 straightens up kitchen, talks with observer.
21:25	+/− [37 min]	−/+ [1 min]	P1 removes P2 from net, takes him to urinate. Both get back into net. P1 gets back out of net.
21:30		− [17 min]	P2 gets back out of net, sits with P1.
21:47		+	P2 gets back into net.
21:55		− [7 min]	P2 gets back out of net, goes to find P1.
22:02	+	+	P1 & P2 get into net.
22:06	−/+ [1 min]		P1 gets out of net to look for socks for P2, finds socks, gets back in.
22:10	−/+ [1 min]		P1 gets out of net to close bedroom door, gets back in.
01:45	−/+ [1 min]		P1 gets out of net to get juice for P2, then gets back in & helps P2 drink juice.
01:47	− [5 min]	− [5 min]	P1 takes P2 out of net to urinate.
01:52	+	+	P1 and P2 get back into net.
06:05	− [5 min]		P1 gets out of net, goes outside, opens door to chicken coop to let chickens out.
06:10	+		P1 gets back into net.
06:22			P2 wakes up, looks at P1, sees she is still asleep, stays inside net sitting up.
06:30	−	−	P1 and P2 both get out of net.
**Total minutes outside net from first entry (after 18:30)**	**195 (225)**	**30 (60)**	
**Total number of times net lifted**	**20**

An unanticipated finding from an observational study of bed net use was the number of times people enter and exit their bed nets during the night—as many as 20 times for 1 mother with a young son.

#### Additional Potential Risk Factors

Observations revealed other phenomena that would have been difficult to capture with interviews or focus groups. For instance, observers took detailed notes on sleeping spaces in participating households. These notes revealed that many people slept directly on cane flooring rather than on a bed. The flooring had gaps between the cane staves. Since many houses were built on stilts, this meant mosquitoes could enter the sleeping space from below. A net alone could not provide adequate protection in this setting: An effective malaria prevention intervention would need to help at-risk individuals find a way to protect themselves from below as well as from above. Observers also documented other practices that might increase exposure risk: attending evening church services during peak biting hours, bathing after dark, running small home-based stores where community members came to buy food or basic necessities in the evening hours, and other nighttime activities such as hunting, fishing, or charcoal production. While study participants reported some of these activities during interviews, direct observation allowed the study team to document them more systematically.

## CASE STUDY #3: ASSESSING THE COMPETENCY OF SKILLED BIRTH ATTENDANTS IN 7 COUNTRIES

### Background

About 90% of the 300,000–350,000 annual maternal deaths worldwide are caused by 5 common obstetric complications: postpartum hemorrhage, pregnancy-induced hypertension, obstructed labor, perinatal sepsis, and postabortion complications.^44^^,^^45^ Risk for experiencing one of these life-threatening complications cannot be reliably predicted in advance, but most can be treated successfully if the woman experiencing them has access to basic or comprehensive essential obstetric care delivered by an SBA. For this reason, the World Health Organization (WHO) recommends that all pregnant women be assisted by an SBA during labor and delivery.^46^ Several international organizations have defined the competencies necessary to manage these complications. The observations described below were carried out as part of developing a method to assess these competencies among practicing SBAs in low- and middle-income countries.

### Study Context and Observation Methods

Testing a clinician's competency to manage a complication according to standards requires assessing not only abstract knowledge but also physical or manual ability. Knowledge can be measured using a written exam, but the only way to assess manual skill is by watching someone perform a task to see whether she or he does it correctly. Assessing skills on actual patients, however, is problematic. Ethically, an observer qualified to evaluate clinical competency would need to stop observing and intervene before allowing an insufficiently skilled provider to endanger a patient's life or well-being. Moreover, even common obstetric complications are relatively rare. This makes it impossible to assess the skill of more than a handful of providers using actual patients.

While knowledge can be measured using a written exam, the only way to assess manual skill is by watching someone perform a task.

The observations discussed here were designed to test SBA competency at performing 4 critical procedures. The first 3 procedures—active management of the third stage of labor (AMTSL), manual removal of the placenta, and bimanual uterine compression—are performed to prevent or control postpartum hemorrhage in a mother who has just given birth. The fourth, neonatal resuscitation with an Ambu bag, is used to treat neonatal asphyxia. The project, eventually carried out in Benin, Ecuador, Jamaica, Kenya, Nicaragua, Rwanda, and Tanzania, used expert obstetrician/gynecologists and pediatricians from host countries as observers. SBAs being assessed performed each procedure on an anatomical model (Gaumard S500 Advanced Childbirth Simulator and Simulaids Sani-Baby CPR mannequin or Gaumard S320 Newborn Airway Trainer); observers assessed competency using a structured step-by-step checklist (Supplement 3).^27^^,^^28^

### Critical Findings

**FIGURE 4:**
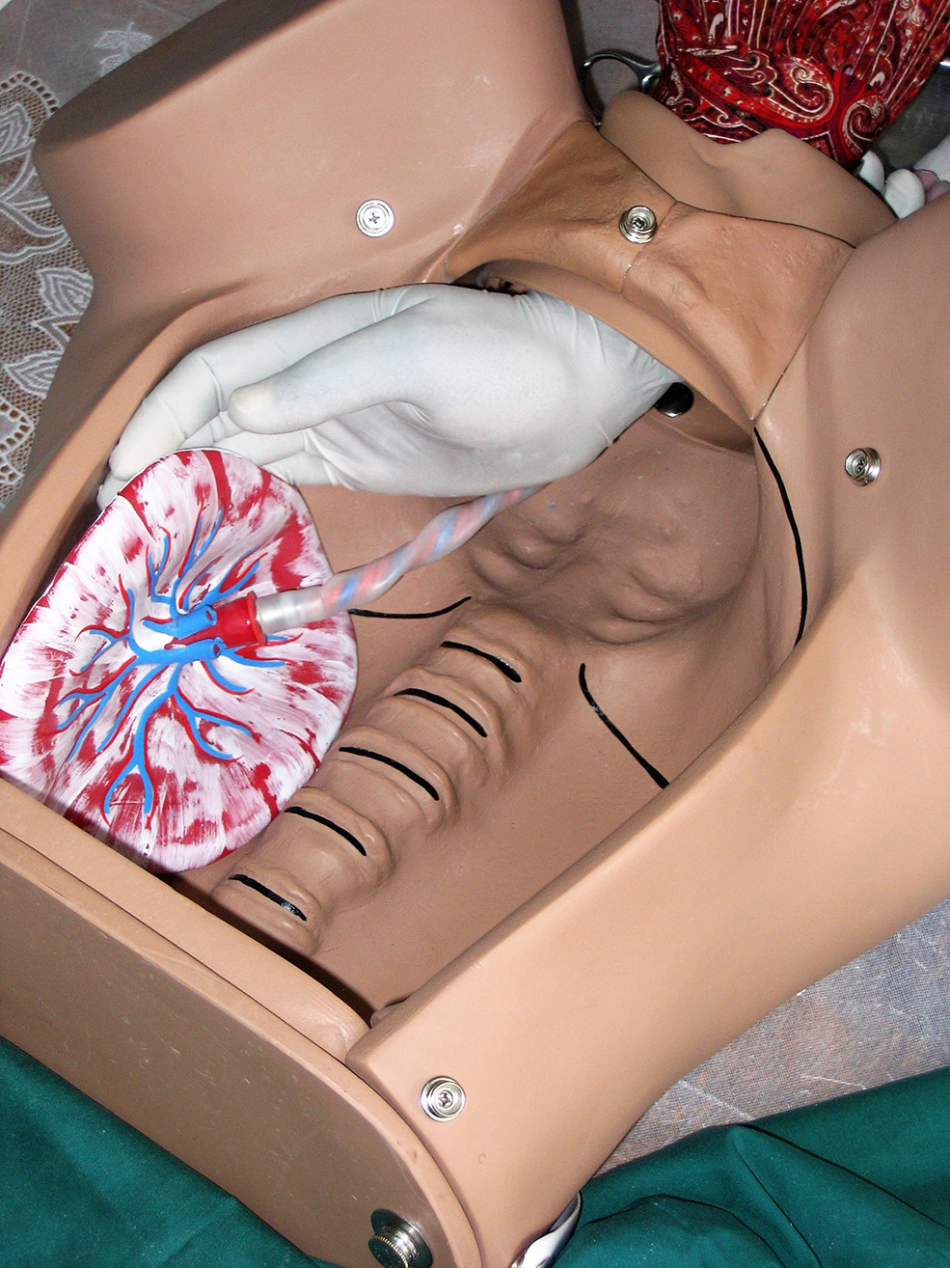
Demonstration of the Correct Hand Position for Manual Removal of a Retained Placenta on an Anatomical Model © 2006 Steven Harvey

Correct hand position and movement are essential to successfully performing all 4 tasks. Controlled cord traction, an elective component of AMTSL, requires exerting a gentle downward pull on the umbilical cord with one hand while using the other to prevent uterine inversion by applying counter-traction just above the pubic bone.^47^ In case of retained placenta, manual removal requires inserting the hand through the vaginal canal and using a gentle lateral motion to detach the placenta intact, leaving no fragments that could provoke continued bleeding or cause sepsis. Figure 4 shows an expert observer demonstrating manual removal with the Gaumard Advanced Childbirth Simulator. The open abdominal cavity allows the observer to assess the technique of the SBA being observed. Some SBAs might be able to describe these or similar procedures, but even a precise detailed description would not necessarily indicate ability to perform them.

**FIGURE 5:**
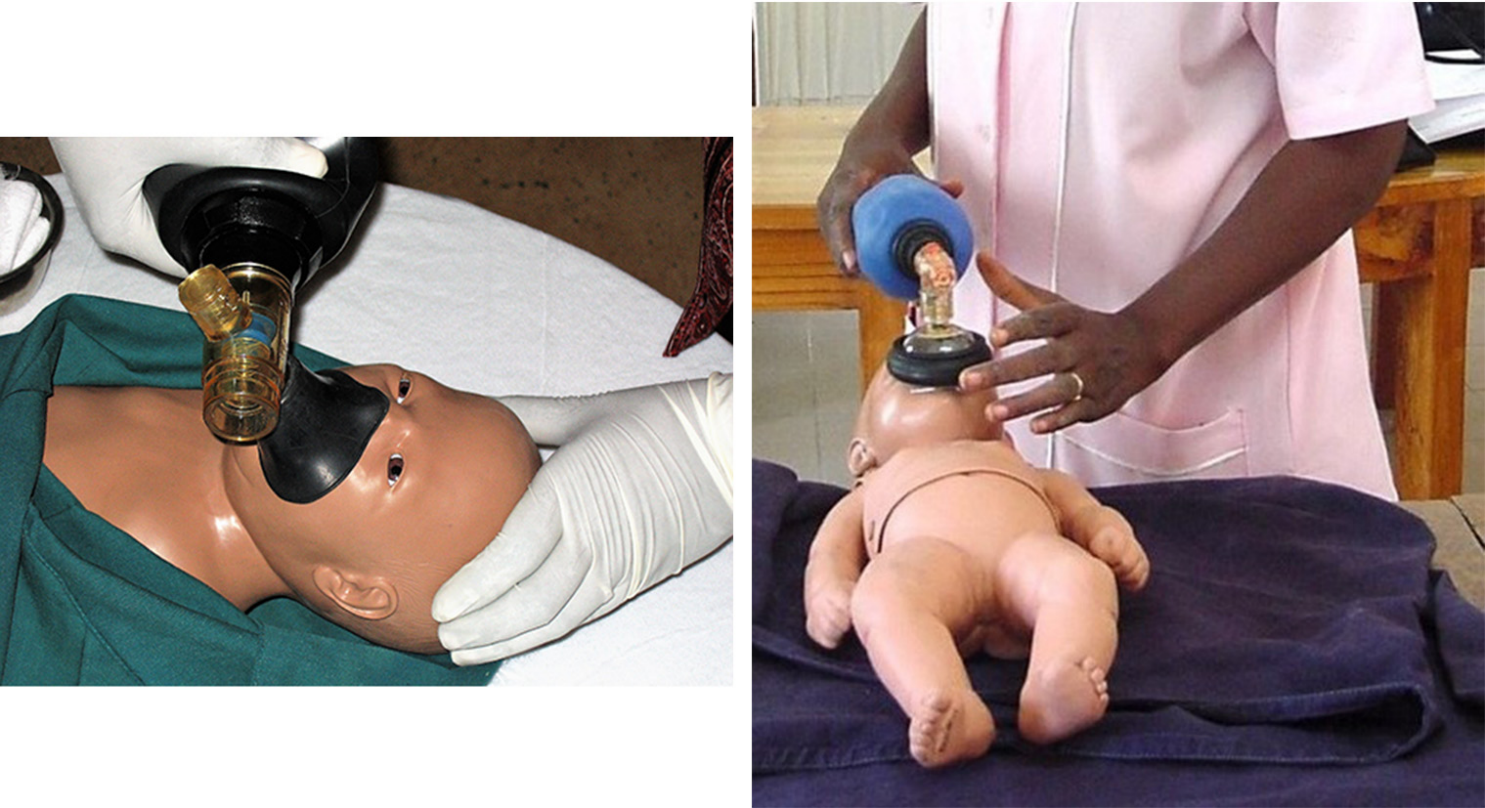
Neonatal Resuscitation With an Ambu Bag: Correct vs. Incorrect Positioning Left: Correct positioning of mask, bag, and newborn's head to achieve a good seal, with bag perpendicular to the newborn's body. © 2006 Steven Harvey. Right: Incorrect positioning, with bag parallel with the newborn's body, making it more difficult to achieve a good seal. © 2002 Steven Harvey.

Observations across the 7 study countries revealed the following:

- Though AMTSL is commonly included in national standards for managing uncomplicated delivery, most SBAs did not know how to perform controlled cord traction.
- Similarly, most SBAs could not demonstrate the correct hand positions for carrying out the manual removal of a retained placenta. Although bimanual uterine compression is a relatively simple procedure requiring no instruments or equipment, virtually no SBA was familiar with it.
- Neonatal resuscitation with an Ambu bag—in addition to requiring a neonatal-sized bag, valve, and mask, which many health facilities lack—requires the person performing it to place the mask over the newborn's mouth and nose and position his or her hand over the mask correctly to achieve a proper seal. Failure to do so can result in air escaping out the sides of the mask rather than entering the newborn's lungs. Proper head position is also critical to ensuring that the newborn's airways are open, not blocked. In Figure 5, the left image shows correct positioning of the bag, mask, and head while the right image shows incorrect positioning: Placing the bag vertically with respect to the newborn's body makes it more difficult to achieve a good seal. Both newborn mannequins used in the study were designed so that the mannequin's chest would rise when ventilated properly, similar to the chest of an actual newborn receiving correct ventilation. This allowed both the observer and the SBA to determine if the SBA was performing the procedure correctly.

Using checklists adapted to each country's norms, observation also enabled the study team to assess whether SBAs followed prescribed infection prevention guidelines including handwashing, gloving, and post-procedure decontamination. Participating SBAs were provided with all necessary supplies and equipment. At the beginning of each assessment, the observer instructed each participant to “begin by preparing yourself, the equipment, and the patient,” then noted if the SBA proceeded in accordance with norms. At the end, the observer similarly instructed each participant to “please tell me what more you would do or ask someone else to do once you have finished the procedure.”

It's tempting to classify this research as *summative* since its initial objective was to assess existing health worker skills. But it was also *formative*, because the results helped shape interventions: In the short term, observers offered feedback and retraining to each participant, and sometimes—when many participants had a particular weakness in common—to the entire group. In the longer term, findings have influenced training programs and assessment methods in participating countries and around the globe.

## CASE STUDY #4: ASSESSING CHW ABILITY TO USE MALARIA RAPID DIAGNOSTIC TESTS IN ZAMBIA

### Background

For decades leading up to the early 2000s, malaria in sub-Saharan Africa was diagnosed presumptively: Anyone with a fever was presumed to have malaria and treated with antimalarials. This practice developed because the supply of both microscopes and trained microscopists was too limited to diagnose more than a tiny fraction of febrile patients. In addition, first-line antimalarial drugs were cheap and adverse effects negligible, so presumptive treatment involved minimal cost and risk. After introduction of artemisinin combination therapy as first-line treatment for malaria starting around 2004, WHO recommended parasite-based diagnosis first for adults and older children, then for all suspected cases of malaria regardless of age.^48^ Malaria rapid diagnostic tests (RDTs) make parasite-based diagnosis possible even at health facilities with no laboratory, microscope, or microscopist. In many areas, however, febrile patients seek treatment at the community level without ever visiting a health facility. The observations described in this case study were carried out to determine whether volunteer community health workers (CHWs) could use RDTs safely and accurately and, if so, what sort of training materials they needed.

### Study Context and Observation Methods

Based on focus group discussions with Zambian CHWs, the study team designed a job aid and brief training curriculum. We used structured observation to pilot test these materials. Study team members observed 79 CHWs prepare 3 RDTs each and recorded the results on a 16-item checklist (Supplement 4).^24^^,^^25^

### Critical Findings

**FIGURE 6:**
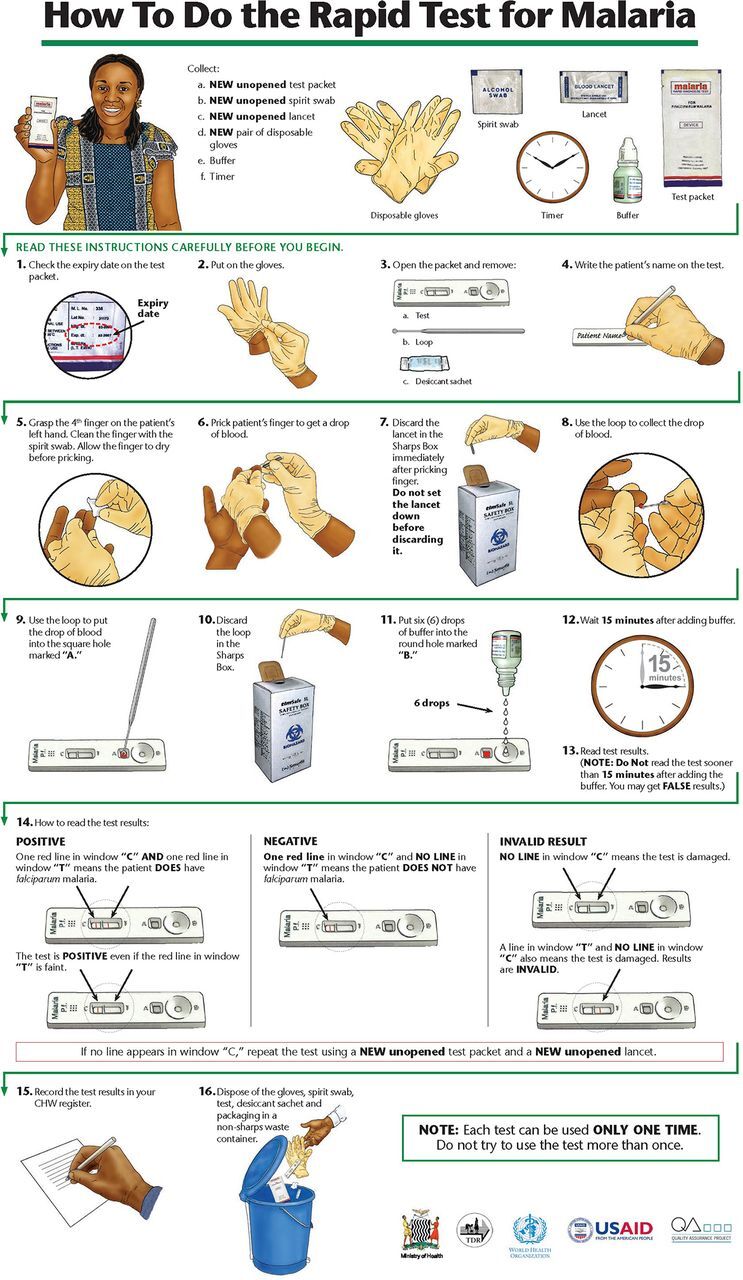
Malaria Rapid Diagnostic Test Job Aid A job aid for community health workers lists at the top all supplies and equipment that the worker needs to assemble prior to conducting a rapid diagnostic test for malaria.

- Malaria RDTs require using a sterile lancet to draw a finger-stick blood sample, a procedure that is second nature to many professional health workers. Due to concerns about HIV and other blood-borne diseases, however, most African CHWs were prohibited from taking finger-stick blood samples. The Zambian Medical Council authorized the practice for this study, but few participating CHWs had ever taken a sample or used a lancet. During training, observers noticed that instead of drawing blood with a quick stab—the preferred approach—many CHWs set the point of the lancet on the patient's fingertip, then pushed it into the skin. Participants explained they were doing this for fear that stabbing would cause the patients too much pain, but the effect was just the opposite: Pushing was more painful. In addition, it often produced too little blood, thus necessitating a second, third, or even fourth finger prick. Observing this made clear that CHWs needed specific training on proper lancet technique. The study team subsequently developed a training module demonstrating how to extract sufficient blood with a single prick. Improved CHW technique reduced patient discomfort and increased testing quality.
- Watching CHWs transfer blood from fingertip to test cassette yielded a similar revelation. The project RDT came packaged with a loop-shaped blood transfer device designed to collect a 5 μl film of blood across the width of the loop. CHWs did the finger prick with the ball of the patient's finger facing up, then tried to collect the drop from above. This often conveyed too little blood to the test cassette even after multiple tries. Noting this, an experienced observer suggested pricking the finger, rotating the patient's hand 180°, then collecting the drop from underneath with the ball of the finger facing down. In most cases, this made it possible to collect and transfer the precise volume of blood required on the first attempt.
- A key concern related to blood safety was correct disposal of the blood-contaminated lancet. To minimize danger to patients, CHWs, and the community, the research team distributed sharps boxes to all participating CHWs and instructed them to deposit the used lancet into the sharps box immediately after pricking the patient's finger. Setting down the used lancet prior to disposal heightens risk of finger-stick injuries. Observers noticed that positioning the sharps box appropriately made immediate disposal convenient: For a right-handed CHW, this meant placing the sharps box on the right side of the work space, and vice versa for a left-handed CHW. Placing the box on the opposite side of the CHW's dominant hand forced the CHW to reach across both his or her own body and that of the patient. This made handling the used lancet more risky and immediate disposal more difficult.
- Assembling supplies prior to conducting a test revealed a similar issue. Most CHWs work from home. Lacking permanent work space, they take out their supplies and then put them away again for each patient. In pilot testing, observers noticed that CHWs would often forget one or more items prior to starting a test. In some cases, this posed only minor inconvenience: CHWs might open a test kit, realize they had forgotten to bring a pen or pencil, and ask the patient to wait while they retrieved one. Other cases presented greater risk: CHWs might open a sterile lancet, realize they had forgotten an alcohol swab or the sharps box, and set the open lancet down on a table while going to retrieve the missing item. This finding led the team to modify the job aid by listing all necessary supplies and equipment at the top and adding an instruction to assemble everything before starting the procedure (Figure 6).
- Watching CHWs provide services from home led to another observational finding: Many CHW homes lack electricity and thus have poor-quality artificial lighting. This fact can affect the accuracy of test interpretation when RDTs are prepared inside, especially after dusk or during inclement weather. The RDT's positive test line—indicating that a patient is infected with malaria—can often be quite faint. With inadequate artificial lighting inside and insufficient natural light outside, a CHW could easily misread a faint positive result as negative, thus leaving an infected patient untreated. Realizing this led to added emphasis during training that positive lines are sometimes quite faint and that CHWs should read results in the brightest light possible to avoid missing a faint positive.

## DISCUSSION

Observation produced novel insights in the case studies just described, but how do you decide when observation might be valuable or even essential for your intervention or study? To answer this, it's useful to think in terms of categories of events or processes. Among others, these might include mechanical skills, health service delivery processes, effects of the built environment, and habitual practices that people would have difficulty articulating, sometimes known as “tacit knowledge.”^49^^,^^50^

Observation can produce novel insights, but how do you decide when it might be valuable or even essential for your intervention or study?

### Mechanical Skills

The SBA and RDT case studies both illustrate the value of observation to understanding mechanical skills, including critical details such as the correct hand position needed to effectively carry out a lifesaving obstetric or neonatal intervention. Manual removal of a retained placenta or resuscitation of an asphyxiated newborn are two examples. Although lancet technique, sharps box position, or collecting blood with the fingertip facing up or down might seem like minute details when preparing an RDT, they can make the difference between effective, efficient, safe practices and practices that lead to incorrect results or endanger the patient, the health worker, or the community. Observation in these cases is critical not only to diagnose lapses but also to identify interventions that can address them. Observation thus led to additional practical training for SBAs and to development of specific training modules and revised job aid illustrations for malaria RDTs. Beyond their specific substantive findings, these two studies highlight the value of observation to understanding both health worker and community behavior.

### Sequential Processes

Many public health interventions involve sequential processes: Not only must each step be performed properly, it must also be performed in the proper order. Again, the RDT case study offers an illustrative example: The study team identified 16 discrete steps necessary to correctly prepare and interpret the test; performing them in the wrong order (e.g., opening the sterile lancet before cleaning the finger with an alcohol swab) or the wrong way (depositing the blood drop where the buffer solution is supposed to go) could compromise test accuracy or patient or health worker safety. The observation checklist (Supplement 4) enabled the team to determine the proportion of health workers who completed all steps correctly, identify specific steps where health workers had problems, and modify training to address the problems observed. Greenland et al. used a similar approach in Zambia to determine what proportion of caregivers of young children with diarrhea could prepare oral rehydration solution correctly.^51^ Hurley et al. used a combination of structured and unstructured observations to track the flow of pregnant women through antenatal care in Mali and better understand why many completed their visits without receiving intermittent preventive treatment for malaria in pregnancy (IPTp) or received it without any information about the purpose of IPTp.^52^ Hermida et al. found observation to be more accurate than patient exit interviews or medical record review for assessing facility-based provider adherence to standards of care for acute lower respiratory infection, diarrheal disease, and family planning counseling.^53^ For this reason, observation is often a key component of quality improvement research.^53^^,^^54^ In sum, observation can be an invaluable tool for documenting the necessary steps in a process, identifying where breakdowns occur, and thus pinpointing where intervention is needed. This type of analysis can be useful at the household, community, and health facility levels.

### Understanding the Built Environment

The built environment—and sometimes its relationship to the natural environment—can significantly affect disease risk, health service delivery, and the feasibility of health interventions. The *Campylobacter* study setting consists of dusty desert hills where water is scarce and rain nonexistent (natural environment). Since the poorest people live at the top of those hills with neither wells nor piped water (built environment), many families struggle to provide water for themselves. Water for corralled birds becomes, at best, a secondary priority. Observing the difficulty of obtaining water helped study team members better understand owners' concerns about the effect of corralling on birds' health. Wind (natural environment) combined with open storage of concentrated chicken manure cleaned from the corrals (built environment) turned out to be one form of continued contact between humans and *Campylobacter* despite corralling.

The built environment was likewise a critical aspect of the bed net study. The structure of a typical bed in the study setting—no mattress and gaps between the wooden or bamboo slats that allowed mosquitoes to bite from underneath—might never have occurred to public health practitioners, most of whom presumably sleep in beds with mattresses. Even had it occurred to them, they would not have been able to collect systematic data on bed configurations without observation. Thus, observation revealed one potential limitation of bed net efficacy in the study setting. This, in turn, revealed a necessary component of any improvement intervention: figure out how to block the gaps between flooring that allowed mosquitoes to enter.

Systematically observing the built environment can be revealing in many settings. By documenting patient flow at health centers and hospitals, maternal health researchers from the Quality Assurance Project helped explain why women arriving with an obstetric complication might encounter significant, sometimes life-threatening, delays before seeing a clinician.^55^^–^^58^ Observing both the size of rooms in a house and their use for multiple purposes (sleeping at night, running a small retail shop during the day) helped explain why some households in Ghana were reluctant to permanently install bed nets over their sleeping spaces and why, in some cases, residents preferred conical nets to rectangular.^59^ Observing the dim lighting in CHWs' houses helped explain why CHWs might miss weak positive RDT results and why training programs needed to emphasize the importance of reading test results under bright light.^25^ Many U.S. researchers have used observation to study the relationships between built environment, physical activity, available food choices, and chronic diseases such as obesity and diabetes.^60^^–^^63^ As with the discussion of sequential processes above, it is worth reiterating that observations related to the built and natural environments can be useful at the household, community, and health facility levels.

### Habitual Practices and Tacit Knowledge

In any setting, people perform a variety of routine activities, the procedures for which they learned at some point in the past, committed to memory, and carry out automatically, almost as if by instinct. Because these activities are habitual, those who perform them often have difficulty articulating the step-by-step process and even come to think of that process as self-evident. Collecting a finger-stick blood sample is a case in point. A health care provider who has done it many times considers it second nature and wonders why a novice finds it so difficult. Observation reveals that the process involves numerous steps: assemble all the supplies before starting, swab the fingertip with alcohol, wait for it to dry, massage the finger to work the blood up into the fingertip, open the sterile lancet, puncture the fingertip with a quick stab, orient the fingertip with the blood drop in the optimum position for the particular blood collection device being used, etc. The experienced provider has internalized all this and performs it without needing to think. The novice may fail to massage the finger, stab too timidly and thus extract too little blood, or orient the fingertip in a less than optimal position and thus collect too little blood, or too much. Observing both expert and novice helps distinguish the differences and thus determine what training the novice requires.

People who perform habitual activities often have difficulty articulating the step-by-step process and even come to think of that process as self-evident.

The *Campylobacter* study provides additional examples: Interview or focus group participants might fail to mention the many points of contact between children and birds either because they knew the intervention was meant to separate the two (courtesy bias) or because the types of contact were so commonplace as to seem unworthy of mention. Observing children play with birds, feed and water them, collect eggs, and clean corrals provides tangible evidence that those designing public health interventions should take into account both human nature (children like to play with animals) and economic and cultural practices (even a very young child may be assigned household chores; parents may view learning to raise animals as a key life skill). Cumulative findings from these observations contributed to a conclusion that the intervention was unlikely to succeed, a conclusion confirmed by subsequent research demonstrating that corralling, instead of decreasing risk of *Campylobacter*-associated diarrhea in children, actually doubled it.^38^

The bed net study also provides examples: Absent observation, as noted above, public health practitioners might not have thought to ask about bed design. Conversely, mentioning bed design—an aspect of daily existence so routine as to pass virtually unperceived—might never have occurred to a member of the at-risk population. Had interviewers thought to ask, net occupants might also have mentioned that they enter and exit their nets more than once per night, but it is unlikely that they could have reported very precisely the number of entries and exits, the amount of time the net was lifted, or the amount of time different occupants spend outside the net. Observation made it possible to quantify this phenomenon much more systematically.^43^

After validating the method, Gittelsohn used structured mealtime observations to estimate differences in caloric and micronutrient intake between men, women, and children in lowland south-central Nepal.^64^^–^^66^ It is unlikely that parents would have been able to provide such detailed information about intra-household food allocation. Bentley et al. used structured observation during formative research to document child feeding practices prior to a nutritional intervention to improve infant growth and development in Andhra Pradesh, India.^10^ Brummell used observation to discover tacit knowledge related to the prognosis of patients suffering cardiac arrest and whether to attempt resuscitation in 2 UK hospital emergency departments.^67^ Huot and Laliberte Rudman, who used participant observation to learn about the daily routines of refugees in Canada, explain why observation can be so important for understanding habitual phenomena^68^:

*The tacit nature of daily occupation can make the details involved in participation difficult to verbalize because respondents may not have reflected upon their occupational engagement in such detail, or may assume that such “minutia” may not be relevant for research.*

This statement could be extended to many areas of health at individual, household, community, and facility levels. Often observation, used together with more common methods like interviews or focus groups, is the only way to make such tacit knowledge explicit.

### Triangulating Observation Data With Data From Other Methods

In both the case studies described here and many of the examples cited, researchers used observation together with other methods to achieve a more complete picture of a setting, practice, or intervention. Using observation to triangulate information gathered from interviews or focus group discussions can bring to light differences between what people say they do (reported behavior) and what people actually do (observed behavior). In some cases, this may reveal social desirability bias: People over- or under-report a particular behavior because it violates what they perceive to be social norms. Hygiene studies, for instance, have often found that people over-report handwashing at critical times; observation shows much lower levels.^69^^,^^70^

Using observation to triangulate information from interviews or focus group discussions can bring to light differences between what people say they do and what they actually do.

There is no Peruvian data on reported ITN use that we can compare to the case study #2 observation. But there is at least a plausible basis for comparison in Ghana: Nighttime observation of net use in Northern and Upper West Regions found that only 17% of the population used a net at any time during the night.^71^ In a malaria indicator survey of the same 2 districts, 51% and 54% of the population reported sleeping under a net.^72^ The numbers are not directly comparable for many reasons, so these differences should be interpreted with caution. The observation study is based on a small purposive sample, the survey on a population-based representative sample; the data were collected in different years and at different times of year. But the wide gap suggests a considerable difference between reported and actual net use. Also, for the observation sample, we know when each individual entered and exited his or her net and how long individuals spent protected versus unprotected. All we know from the survey is that the individual reported sleeping under the net at some point during the night—we have no idea for how long.

Triangulation may also reveal that a word, phrase, or concept means something different to participants than to the researcher. The possibility, in the *Campylobacter* study, that participants who reported keeping their birds in the corral “all the time,” really meant “all the time except for certain specific seemingly obvious times” is one example. Had we employed only interviews in that study, we would likely have concluded—incorrectly—that birds were never loose. Had we employed only observation, we would likely have concluded that birds were loose 20% of the time—more accurate, but not the whole story. Only the *combination* revealed the differences in meaning and their conflicting unspoken assumptions.

### Observation and Reactivity

A key objection to observation is that it leads to reactivity: Those under observation may change their behavior because they know they are being observed. However, this problem is not unique to observation: People also change their behavior when they are being studied in other ways. Survey and interview respondents may answer questions based on what they think society (social desirability bias) or the interviewer (courtesy bias) expect of them. Observer expectancy effect refers to how an observer can shape behavior—deliberately or subconsciously—by providing subtle nonverbal cues such as slight changes in facial expression. The Hawthorne effect was named for a study in which factory workers from both intervention and control groups became more productive because they knew that researchers were testing possible interventions (such as better lighting) to improve productivity. More detailed definitions are beyond the scope of this article but can be found in many social science references.^73^^–^^76^

In one example of reactivity, P.V. Ram and colleagues found evidence of a 35% increase in handwashing when an observer was present compared with when there was no observer and handwashing was detected by a motion sensor hidden within a bar of soap.7^77^ But while reactivity often does occur, researchers can measure and adjust for it.^17^ Reactivity also diminishes with time: The longer amount of time or the greater number of times people are observed, the less likely they are to react to an observer's presence.^78^^–^^80^ Ram's study concluded that their findings “call into question the validity of structured observation details because it appears that a majority of participating caregivers substantially altered their behavior in the presence of an observer.” But the study included only 1 observation per household. Had Ram's team observed each household multiple times and waited until household members became accustomed to the observer's presence, their results might have been different.

Ram and her colleagues have a point that in some cases a less invasive technological method might be preferable to observation. For example, studies exploring household use of cleaner cookstoves to reduce indoor air pollution often use temperature sensors (called stove use monitors or SUMs) to track which stove is being used when and for how long.^81^^,^^82^ At least one recent study reports that combining observation and SUMs data provides a more accurate picture than SUMs data alone.^83^

Moreover, reactivity is often unrelated to the focus behavior. In the bed net study, we identified 339 instances of reactivity across 60 observations using the broadest possible definition: any interaction whatsoever between the observer and any member of the observed household. Of these 339 instances, only 2 were directly related to the behavior of interest: protecting against mosquito bites.^17^ In a similar way, John Schnelle and colleagues found that observations did not change provider treatment of nursing home residents in the United Kingdom.^84^

Another way to control reactivity is through unannounced spot checks similar to those we used in case study #1. Nazmul Chaudhury and colleagues used this method to chronicle the degree of health worker and teacher absenteeism in health facilities and primary schools in Bangladesh, Ecuador, India, Indonesia, Peru, and Uganda.^85^ In his classic article about nighttime observations among the Samukundi Abelam, Richard Scaglion describes how he used spot checks to document time allocation within this Papua New Guinea ethnic group.^86^ Scaglion admits, however, that he was not always able to maintain the element of spontaneity that spot check observations are meant to provide:

*… it is not easy for an anthropologist in the field to come upon an Abelam unawares. Since I did not want to record “greeting anthropologist” as a frequent activity when people were first observed, I often had to reconstruct what they were doing immediately before I arrived.*

## CONCLUSION

In sum, observation can be an essential tool in formative research. As a stand-alone method, it can measure phenomena not measurable by any other method. In combination with interviews or focus groups, it can suggest questions to be posed through these other methods. It can also triangulate findings from other methods, reveal potential differences between reported and observed behavior, and thus help assess social desirability bias. Given these benefits, observation—either alone or in combination with other methods—is something both investigators and program managers should consider when undertaking formative research.

## Supplementary Material

17-00328-Harvey-Supplement4.doc

17-00328-Harvey-Supplement3.doc

17-00328-Harvey-Supplement1.docx

17-00328-Harvey-Supplement2.docx

## Notes

**Peer Reviewed**

**Funding:** Funding for case study #1 was provided by the Thrasher Research Fund (award 02813-1). Funding for case study #2 was provided by the US Agency for International Development (USAID) under Grant Number 527G001000070. Case study #3 was supported by the Quality Assurance Project under contracts number HRN-C-00-96-90013 and GPH-C-00-02-00004-00 with the United States Agency for International Development (USAID). Funding for case study #4 was provided by the Australian Agency for International Development (AusAID), the WHO Special Programme for Research and Training in Tropical Diseases (TDR), and the United States Agency for International Development (USAID) under the Quality Assurance and Workforce Development Project at University Research Co., LLC (contract number GPH-C-00-02-00004-00). Conclusions and opinions are the sole responsibility of the author and do not necessarily reflect the views or policies of the funders.

**First Published Online:** May 23, 2018
