# Supplementary material for: Observe Before You Leap: Why Observation Provides Critical Insights for Formative Research and Intervention Design That You'll Never Get From Focus Groups, Interviews, or KAP Surveys
Source: Glob Health Sci Pract. 2018 Jun 27;6(2):299–316. doi: 10.9745/GHSP-D-17-00328 (PMC6024634; doi:10.9745/GHSP-D-17-00328)
Supplement: 17-00328-Harvey-Supplement4.doc [file 17-00328-Harvey-Supplement4.doc]

| ***Form 1: Observation of Paracheck Cassette RDT Use, Zambia 2006*** | | | | | | | | | | | | | | | | | | | |  | | |  |
| --- | --- | --- | --- | --- | --- | --- | --- | --- | --- | --- | --- | --- | --- | --- | --- | --- | --- | --- | --- | --- | --- | --- | --- |
| **PLEASE FILL IN FORM COMPLETELY AND LEGIBLY. USE BLOCK CAPITAL LETTERS.** | | | | | | | | | | | | | | | | | | | | CHW # (for internal use only) | | |  |
| Observer | **Date observed** | | | | | | | | District | | | | Sex | | Age | Education (yrs ***completed***) | | Malaria tx experience? | | | Ever used RDTs? | | |
|  |  |  |  |  |  |  |  |  |  |  |  |  |  | [1] M |  |  | [1] Some primary |  | [1] Yes | |  | [1] Yes | |
|  |  |  |  |  |  |  |  |  | Health facility name | | | |  |  |  |  | [2] Complete primary |  | [2] No | |  | [2] No | |
| CHW’s given name | Day | | Month | | Year | | | |  |  |  |  |  | [2] F |  |  | [3] Some secondary |  | How many months? | |  | For how many months? | |
|  |  |  |  |  |  |  |  |  | Round (tick one) | 1 | 2 | 3 |  |  |  |  | [4] Complete secondary or more |  |  |  |  |  |  |

| ***Table A: Observation of three RDT tests*** | | | | | | | | | | | |
| --- | --- | --- | --- | --- | --- | --- | --- | --- | --- | --- | --- |
| **Observation Number** | **1** | | | **2** | | | **3** | | | **Comments** | |
| **Was this test done on a real patient? *Circle the correct answer: 1=Yes 2=No*** | 1Y | 2N |  | 1Y | 2N |  | 1Y | 2N |  |  | |
| **Was patient febrile? *Circle the correct answer: 1=Yes 2=No 3=Not applicable (if not a real patient)*** | 1Y | 2N | 3 | 1Y | 2N | 3 | 1Y | 2N | 3 |
| ***For each step below, circle* 1 *if the CHW performed the step correctly, circle* 2 *if the CHW performed the step incorrectly, circle* 3** *if* ***the CHW skipped the step*** | | | | | | | | | |
| 1. **Assemble new test packet, buffer, swab, lancet, timer & gloves.** | 1 | 2 | 3 | 1 | 2 | 3 | 1 | 2 | 3 |  |  |
| 1. **Check expiry date on test package to make sure test is still valid.** | 1 | 2 | 3 | 1 | 2 | 3 | 1 | 2 | 3 |  |  |
| 1. **Put on new pair of gloves.** | 1 | 2 | 3 | 1 | 2 | 3 | 1 | 2 | 3 |  |  |
| 1. Remove contents of test packet. | 1 | 2 | 3 | 1 | 2 | 3 | 1 | 2 | 3 |  |  |
| 1. Write patient’s name on cassette. | 1 | 2 | 3 | 1 | 2 | 3 | 1 | 2 | 3 |  |  |
| 1. Select 4^th^ finger of left hand; clean it with spirit swab; allow it to air dry. | 1 | 2 | 3 | 1 | 2 | 3 | 1 | 2 | 3 |  |  |
| 1. Using a sterile lancet, puncture the finger. | 1 | 2 | 3 | 1 | 2 | 3 | 1 | 2 | 3 |  |  |
| 1. **Discard lancet in sharps bin immediately after pricking finger. Do not set it down first.** | 1 | 2 | 3 | 1 | 2 | 3 | 1 | 2 | 3 |  |  |
| 1. **Collect film of blood with the enclosed loop making sure to fill the loop completely.** | 1 | 2 | 3 | 1 | 2 | 3 | 1 | 2 | 3 |  |  |
| 1. **Using the loop, blot blood on the pad in sample well A.** | 1 | 2 | 3 | 1 | 2 | 3 | 1 | 2 | 3 |  |  |
| 1. **Discard the loop in the sharps box.** | 1 | 2 | 3 | 1 | 2 | 3 | 1 | 2 | 3 |  |  |
| 1. **Dispense six drops of clearing buffer into well B.** | 1 | 2 | 3 | 1 | 2 | 3 | 1 | 2 | 3 |  |  |
| 1. **Wait 15 minutes before reading results.** | 1 | 2 | 3 | 1 | 2 | 3 | 1 | 2 | 3 |  |  |
| 1. **Read test results correctly.** | 1 | 2 | 3 | 1 | 2 | 3 | 1 | 2 | 3 | **Did CHW know correct expiry of last test?** | |
| 1. Record results in CHW register. | 1 | 2 | 3 | 1 | 2 | 3 | 1 | 2 | 3 |
| 1. Dispose of gloves, wrappers, alcohol swab, loop, desiccant & cassette in non-sharps container. | 1 | 2 | 3 | 1 | 2 | 3 | 1 | 2 | 3 |
| **Row Total (Add the number of “1s” in the left-most column of each round):** |  | /16 |  |  | /16 |  |  | /16 |  | **Y** | **N** |

| ***Table B: Interpretation of RDT results*** | | | | | | | | | | |
| --- | --- | --- | --- | --- | --- | --- | --- | --- | --- | --- |
| **Battery #** | **1** | **2** | **3** | **4** | **5** | **6** | **7** | **8** | **9** | **10** |
| **Test result** | *(Write an* ***“X”*** *in the appropriate box below each test number to indicate if the BHW interprets the test result as “Positive” “Negative” or “Invalid”)* | | | | | | | | | |
| **Positive** |  |  |  |  |  |  |  |  |  |  |
| **Negative** |  |  |  |  |  |  |  |  |  |  |
| **Invalid** |  |  |  |  |  |  |  |  |  |  |
