# Supplementary material for: Observe Before You Leap: Why Observation Provides Critical Insights for Formative Research and Intervention Design That You'll Never Get From Focus Groups, Interviews, or KAP Surveys
Source: Glob Health Sci Pract. 2018 Jun 27;6(2):299–316. doi: 10.9745/GHSP-D-17-00328 (PMC6024634; doi:10.9745/GHSP-D-17-00328)
Supplement: 17-00328-Harvey-Supplement3.doc [file 17-00328-Harvey-Supplement3.doc]

| **Date:** |  |  |  |  |  |  |  | |  | | **Province:** | | | | | |  | | | | | | **District:** |  |
| --- | --- | --- | --- | --- | --- | --- | --- | --- | --- | --- | --- | --- | --- | --- | --- | --- | --- | --- | --- | --- | --- | --- | --- | --- |
|  | dd | | mm | | yyyy | | | | | |  | | | | | |  | | | | | |  |  |
|  | | | | | | | | | | | | | | | | | | | | | | | | |
| **Participant study ID No.** | | | | | | | |  | |  | | - | |  |  |  | |  | |  |  | **Evaluator:** | |  |
|  | | | | | | | | | | | | |  | | | | | |  | | | | | |

**Instructions to the Observer**

*Please read the following statements aloud EXACTLY AS THEY ARE WRITTEN, without adding or leaving out anything. This will avoid bias and provide each participant with the same orientation.*

**“Good morning/afternoon Dr., Mr., Ms. _____, I am ______. Welcome and thank you for participating. Please make yourself comfortable and don’t feel anxious. I will explain the exercise; please ask me if you have any questions about what you are supposed to do.**

**At this station we will simulate the active management of the third stage of delivery. We will work with mannequins, but please act as if you are treating an actual patient. You should prepare yourself, prepare the patient, and perform the procedure and subsequent tasks exactly as you would with a real patient.**

**You are midway through conducting a vaginal delivery. There have been no complications. As you can see** *[observer: point to pelvic mannequin]***, only the baby’s head has been delivered so far. Your task is to finish delivering the baby and then carry out active management at the appropriate time.**

Assume that you are in a rural health center or small district hospital. You have only basic equipment available. If you need help, I can act as your assistant; you may ask me to do anything you would ask another staff/person to do during an actual delivery. While you work, please explain to me verbally everything you are doing—even details that you might think are minor or insignificant.

**Do you have any questions?**

*Observer: answer any questions the participant might have. Once the participant has no more questions to ask say:*

You may begin whenever you are ready.

| **A. Completing Delivery** | **EVALUATION** | | | |
| --- | --- | --- | --- | --- |
|  | **Done correctly** | **Done Incorrectly** | **Not done** | **Not observed** |
| 1. Encourages patient and provides emotional support. |  |  |  |  |
| 2. Prepares 10 units oxytocin (IM) ***EVALUATOR: SEE BELOW NOTE*** |  |  |  |  |
| 4. Finishes delivery and places baby on mothers abdomen (skin-to-skin) |  |  |  |  |

***Note:*** For step 3, if there is no oxytocin available, it is acceptable that the participant says 0.2 mg of ergometrine or prostaglandin. Nevertheless, if the participant mentions ergometrine, he/she MUST mention that the ergometrine is not recommended for women with preeclampsia or eclampsia to earn a satisfactory score. If the participant mentions prostaglandin, he/she MUST mention that this drug must not be administered intravenously to earn a satisfactory score.

*Evaluator: when the participant says that he/she is ready to begin the procedure, tell him/her the following:*

**“Very good, go ahead. Please remember to explain what you are doing and let me know when you have finished.”**

| **B. Procedure: Active Management of the Third Stage of Delivery** | **EVALUATION** | | | |
| --- | --- | --- | --- | --- |
|  | **Done correctly** | **Done Incorrectly** | **Not done** | **Not observed** |
| 1. Palpates the abdomen and rules out presence of another fetus before continuing. |  |  |  |  |
| 1. Within the first minute of the birth, administers 10 units of IM oxytocin. If oxytocin is not available, administers 0.2 mg of ergometrine (NOT in preeclamptic/eclamptic women) or prostaglandins (NO IV). |  |  |  |  |
| 1. Clamps and cuts the umbilical cord. (Clamps near the perineum). |  |  |  |  |
| 1. With one hand, maintains slight tension on the cord and waits for a strong uterine contraction (when the cord stretches, the uterus becomes round). |  |  |  |  |
| 1. During the contraction, applies controlled traction to the cord so as to deliver the placenta: Pulls gently, firmly, and uniformly. |  |  |  |  |
| 1. With the other hand on the pubis, stabilizes the uterus and prevents uterine inversion by applying counter-traction during the controlled pulling of the cord. |  |  |  |  |
| 1. Slowly delivers the placenta, supporting it with both hands. Extracts the membranes gently with lateral movements. |  |  |  |  |
| *Evaluator:* ***“What would you do once the placenta has descended?”*** |  |  |  |  |
| 1. Checks to see if the placenta is whole and intact. |  |  |  |  |
| 1. Checks to see if the tissues are complete. |  |  |  |  |
| *Evaluator:* ***“What would you do if the placenta is intact?”*** | | | | |
| 1. After the inspection of the placenta and membranes, immediately massages the uterine fundus through the abdomen until the uterus contracts. Assures that the uterus does not relax (become atonic); ceases to massage it *after* confirming that the uterus is not softening. |  |  |  |  |
| 1. Carefully examines the woman and repairs cervical or vaginal tears, or repairs the episiotomy. |  |  |  |  |
| *Evaluator: “****What would you do if the placenta is not complete or is not expelled within the first 30 minutes?”*** | | | | |
| 1. If the placenta does not descend during the first 30-40 seconds of controlled traction of the umbilical cord, stop the cord traction and wait for the next contraction to try again. |  |  |  |  |
| 1. If the placenta still doesn’t descend in the first 30 minutes or isn’t expelled intact, ask for help and prepare for additional procedures, probably for manual removal of the placenta. |  |  |  |  |

**CONTINUE ON NEXT PAGE**

*When the participant says that he/she has finished, tell him/her the following:* **“Thank you. Now that you have finished the procedure, please tell me what else you would normally do or ask someone else to do.”** *Observe if he/she mentions the steps listed below. For this final section, it is not necessary that the participant performs each step, but it is required that he/she mentions each step.*

| C. Post-procedure: Active Management of the Third Stage of Delivery | **EVALUATION** | | | |
| --- | --- | --- | --- | --- |
|  | **Done correctly** | **Done Incorrectly** | **Not done** | **Not observed** |
| 1. Submerges both gloved hands in a 0.5% chloride solution. |  |  |  |  |
| 2. Removes the gloves, turning them inside out. |  |  |  |  |
| 3. If the gloves are to be disposed of, participant throws them into a bio-hazard waste receptacle or plastic bag. If the gloves are to be reused, he/she submerges them in a 0.5% chloride solution for 10 minutes to decontaminate them. |  |  |  |  |
| 4. Thoroughly washes hands with soap and water, then dries them. |  |  |  |  |
| 5. Monitors vaginal bleeding and vital signs every 15 minutes for the first 2 hours. |  |  |  |  |
| 6. Makes sure that the uterus remains firmly contracted. |  |  |  |  |
| 7. Encourages the woman to ask questions and provides reassurance. |  |  |  |  |

*Provide appropriate feedback to the participant, answer questions, then say:*

**“Thank you very much. Now you will continue on to the next station to perform the next simulation about a case of postpartum hemorrhage.”**

**Comments on the participant’s performance (please describe any specific errors or problems):**

| **Date:** |  |  |  |  |  |  |  | |  | | **Province:** | | | | | |  | | | | | | **District:** |  |
| --- | --- | --- | --- | --- | --- | --- | --- | --- | --- | --- | --- | --- | --- | --- | --- | --- | --- | --- | --- | --- | --- | --- | --- | --- |
|  | dd | | mm | | yyyy | | | | | |  | | | | | |  | | | | | |  |  |
|  | | | | | | | | | | | | | | | | | | | | | | | | |
| **Participant study ID No.** | | | | | | | |  | |  | | - | |  |  |  | |  | |  |  | **Evaluator:** | |  |
|  | | | | | | | | | | | | |  | | | | | |  | | | | | |

Instructions for the Observer

*Please read the following statements aloud EXACTLY AS THEY ARE WRITTEN, without adding or leaving out anything. This will avoid bias and provide each participant with the same orientation.*

**“Good morning/afternoon. Thank you again for being here today. I am Dr./Mr./Mrs. ______, and I will be observing this next exercise. In the previous exercise, you demonstrated active management of 3^rd^ stage labour. Now we are going to deal with post partum haemorrhage.**

**In this exercise, we will simulate a case of postpartum haemorrhage. Assume that you are in the same health facility as before. Just as in the previous exercise, I can act as your assistant if you need help. You may ask me to do anything you would normally ask of an assistant.**

Another health worker has just attended a normal birth. He performed active management for 30 minutes but the placenta has not delivered. He has called you to perform a manual removal of the placenta. As before, you can assume that appropriate infection prevention measures have already been taken. Please perform the procedure and explain everything you are doing as you go along.

Begin by preparing yourself, the equipment, and the patient. Once you have completed all preparations, please tell me you are ready to begin the procedure.”

| **A. Preparation: Manual Removal of the Placenta** | **EVALUATION** | | | |
| --- | --- | --- | --- | --- |
|  | **Done correctly** | **Done Incorrectly** | **Not done** | **Not observed** |
| 1. Explains to the patient what he/she will do. |  |  |  |  |
| 1. Encourages her to ask questions and listens to what she has to say. |  |  |  |  |
| 1. Provides emotional support. |  |  |  |  |
| *Asks the patient to urinate or catheterizes the bladder, if necessary.* |  |  |  |  |
| 1. Administers anaesthesia or sedatives. |  |  |  |  |
| 1. Begins an IV infusion (normal saline or Ringer’s lactate), adds a 20 unit solution of oxytocin at a rate of 60 drops per minute. |  |  |  |  |
| 1. Administers prophylactic antibiotics in one dose only: 2g IV ampicillin + 500mg IV metronidazole **or** 1g IV cefazolin + 500mg IV metronidazole. |  |  |  |  |

Note: For step 3, if no is oxytocin available, it is acceptable that the participant says 0.2 mg of ergometrine or prostaglandin. If the participant mentions ergometrine, he/she MUST mention that it is not recommended for women with preeclampsia or eclampsia. If the participant mentions prostaglandin, he/she MUST mention that this drug cannot be administered intravenously. Failure to mention these precautions should be marked as “done incorrectly.”

*Evaluator: when the participant says that he/she is ready to begin the procedure, tell him/her the following:*

**“Very well, go ahead. Please explain to me what you are doing at each moment, and tell me** when you have finished the procedure.”

| **B. Procedure: Manual Removal of the Placenta** | **EVALUATION** | | | |
| --- | --- | --- | --- | --- |
|  | **Done correctly** | **Done Incorrectly** | **Not done** | **Not observed** |
| 1. Washes hands and forearms thoroughly with soap and water, then dries them. |  |  |  |  |
| 2. Puts sterile or high-level disinfected gloves on both hands. |  |  |  |  |
| 3. Clamps umbilical cord, gently pulling it until it is parallel with the floor. |  |  |  |  |
| 4. Inserts the fingers of one hand into the vagina and up into the uterine cavity until the placenta is located. |  |  |  |  |
| 5. Places the other hand on top of the abdomen to support the fundus of the uterus, doing counter-traction during the removal so as to prevent inversion of the uterus. |  |  |  |  |
| 6. Delicately slips the ulnar edge of hand between the placenta and the uterine wall, and gradually moves hand from one side to the other, in a lateral continuous movement, until the entire placenta is separated from the uterine wall. |  |  |  |  |
| 7. Slowly removes hand from the uterus, bringing the placenta and membranes with it; meanwhile he/she continues to apply counter-traction to the fundus through the abdomen. |  |  |  |  |
| 8. Examines the uterine surface of the placenta to ensure that it is complete. |  |  |  |  |
| 9. Palpates the interior of the uterine cavity to make sure that all placental tissue has been removed. |  |  |  |  |
| 10. Administers or continues the infusion of 20 units of oxytocin in 1L of normal saline or Ringer’s lactate, at a rate of 60 drops per minute. |  |  |  |  |
| 11. Asks that an assistant massage the fundus of the uterus to stimulate a tonic uterine contraction. |  |  |  |  |
| 12. Carefully examines the woman and repairs any tears to the cervix or vagina, or repairs the episiotomy. |  |  |  |  |

**CONTINUE ON NEXT PAGE**

*When the participant say that he/she has finished, say the following:* **“Very good. Now, please tell me what more you would do or ask that someone else to do once you have finished the procedure.”** *Observe if he/she mentions the steps listed below. For this last section, it is not necessary that the participant perform each step, but it is required that he/she mention each step.*

| C. Post-procedure: Manual Removal of the Placenta | **EVALUATION** | | | |
| --- | --- | --- | --- | --- |
|  | **Done correctly** | **Done Incorrectly** | **Not done** | **Not observed** |
| 1. Submerges both gloved hands in a 0.5% chloride solution. |  |  |  |  |
| 1. Removes the gloves, turning them inside out. |  |  |  |  |
| 1. If the surgical gloves are going to be disposed of, he/she throws them into a bio-hazard waste receptacle or plastic bag. If the gloves are going to be reused, he/she submerges them in a 0.5% chloride solution for 10 minutes so as to decontaminate them. |  |  |  |  |
| 1. Washes hands and forearms thoroughly with soap and water, then dries them. |  |  |  |  |
| 1. Monitors vaginal bleeding and vital signs every 15 minutes during first 2 hours, then every 30 minutes for first 6 hours, even if the patient is stable. |  |  |  |  |
| 1. Makes sure that the uterus remains firmly contracted. |  |  |  |  |
| 1. Explains to the woman what was done and answers her questions. |  |  |  |  |

*Observer: provide appropriate feedback to the participant. Answer any questions, and demonstrate or describe how to correctly perform any steps the participant may have done incorrectly.*

“**Thank you very much Dr./Sr./Mr./Mrs/Ms____ please continue on to the next station.**

**Comments on the participant’s performance (please describe any specific errors or problems):**

| **Date:** |  |  |  |  |  |  |  | |  | | **Province:** | | | | | |  | | | | | | **District:** |  |
| --- | --- | --- | --- | --- | --- | --- | --- | --- | --- | --- | --- | --- | --- | --- | --- | --- | --- | --- | --- | --- | --- | --- | --- | --- |
|  | dd | | mm | | yyyy | | | | | |  | | | | | |  | | | | | |  |  |
|  | | | | | | | | | | | | | | | | | | | | | | | | |
| **Participant study ID No.** | | | | | | | |  | |  | | - | |  |  |  | |  | |  |  | **Evaluator:** | |  |
|  | | | | | | | | | | | | |  | | | | | |  | | | | | |

Instructions to the Observer

*Please read the following statements aloud EXACTLY AS THEY ARE WRITTEN, without adding or leaving out anything. This will avoid bias and provide each participant with the same orientation.*

“Good morning/afternoon. Thank you again for being here today. I am Dr./Mr./Mrs. ______, and I will be observing this next exercise. In the previous exercise, you demonstrated manual removal of the placenta. Now assume that despite all of your efforts, the patient continues to hemorrhage. You are called upon again, this time to perform bimanual uterine compression. Just as with the previous exercise, I am going to ask you to perform the procedure and verbally explain to me everything you are doing, even details that may seem insignificant. Assume that infection control measures have already been taken on the patient. Please begin by describing or carrying out the steps you would take to prepare yourself, the equipment, and the patient. Then tell me when you have finished preparing and are ready to start the procedure itself.”

NOTE:

*If the participant says that he/she does not know the procedure, ask him/her to do the best that he/she can or as far as he/she knows, without worrying about doing it perfectly. Then, show the participant how to do it, following all of the steps indicated below.*

| **A. Preparation: Bimanual Uterine Compression** | **EVALUATION** | | | |
| --- | --- | --- | --- | --- |
|  | **Done correctly** | **Done Incorrectly** | **Not done** | **Not observed** |
| 1. Explains to the patient what he/she will do. |  |  |  |  |
| 1. Encourages her to ask questions and listens to what she has to say. |  |  |  |  |
| 1. Provides emotional support. |  |  |  |  |
| *Asks the patient to urinate or catheterizes the bladder, if necessary.* |  |  |  |  |
| 1. Initiates oxytocin IV infusion or administers ergometrine if the patient is not hypertensive. |  |  |  |  |

*Evaluator: when the participant says that he/she is ready to begin the procedure, tell him/her the following:*

“Very well, go ahead. Please explain to me what you are doing at every step along the way and let me know when you have finished the procedure.”

| **B. Procedure: Bimanual Uterine Compression** | **EVALUATION** | | | |
| --- | --- | --- | --- | --- |
|  | **Done correctly** | **Done Incorrectly** | **Not done** | **Not observed** |
| 1. Washes hands and forearms thoroughly with soap and water, then dries them. |  |  |  |  |
| 2. Puts high-level disinfected or sterile gloves on both hands. |  |  |  |  |
| 3. Introduces one hand in the vagina and makes a fist. |  |  |  |  |
| 4. Places clenched fist in the anterior vaginal fornix and presses against the anterior uterine wall. |  |  |  |  |
| 5. With the other hand, presses deeply into the abdomen behind the uterus, firmly squeezing the uterus between the two hands. |  |  |  |  |
| 6. Maintains the compression until bleeding is controlled and uterus contracts. If bleeding persists, complements or replaces this compression with compression of abdominal aorta and refers immediately to a higher-level facility. |  |  |  |  |
| 7. Verifies that the uterus has contracted. |  |  |  |  |

*When the participant say that he/she has finished, tell him/her the following:* **“Very good. Now, please tell me what more you would do or ask that someone else do once you have finished the procedure.”** *Observe if he/she mentions the steps listed below. It is not necessary that the participant perform each step, but it is required that he/she mention each step.*

| C. Post-procedure: Bimanual Uterine Compression | **EVALUATION** | | | |
| --- | --- | --- | --- | --- |
|  | **Done correctly** | **Done Incorrectly** | **Not done** | **Not observed** |
| 1. Submerges both gloved hands in a 0.5% chloride solution. |  |  |  |  |
| 1. Removes the gloves, turning them inside out. |  |  |  |  |
| 1. If gloves are to be disposed of, he/she throws them into a bio-hazard waste receptacle or plastic bag. If gloves are to be reused, he/she submerges them in a 0.5% chloride solution for 10 minutes to decontaminate them. |  |  |  |  |
| 1. Thoroughly washes his/her hands with soap and water and then dries them. |  |  |  |  |
| 1. Monitors vaginal bleeding every 15 minutes for the first hour and every 30 minutes in the second hour. |  |  |  |  |
| 1. Monitors vital signs. |  |  |  |  |
| 1. Makes sure that the uterus remains firmly contracted. |  |  |  |  |
| 1. Explains to the woman what was done. |  |  |  |  |
| 1. Answers the woman’s questions. |  |  |  |  |

*Observer: provide appropriate feedback to the participant. Answer any questions, and demonstrate or describe how to correctly perform any steps the participant may have done incorrectly.*

“**Thank you very much Dr./Sr./Mr./Mrs/Ms____ please continue on to the next station.**

**Comments on the participant’s performance. Please describe any specific errors or problems. Continue on other side if necessary.**

| **Date:** |  |  |  |  |  |  |  | |  | | **Province:** | | | | | |  | | | | | | **District:** |  |
| --- | --- | --- | --- | --- | --- | --- | --- | --- | --- | --- | --- | --- | --- | --- | --- | --- | --- | --- | --- | --- | --- | --- | --- | --- |
|  | dd | | mm | | yyyy | | | | | |  | | | | | |  | | | | | |  |  |
|  | | | | | | | | | | | | | | | | | | | | | | | | |
| **Participant study ID No.** | | | | | | | |  | |  | | - | |  |  |  | |  | |  |  | **Evaluator:** | |  |
|  | | | | | | | | | | | | |  | | | | | |  | | | | | |

**Instructions to the Observer/Evaluator**

*Please read the following statements aloud EXACTLY AS THEY ARE WRITTEN, without adding or leaving out anything. This will avoid bias and provide each participant with the same orientation.*

### “Good morning/afternoon Dr, Mr., Ms. _____, I am _____.

**“Welcome and thank you for participating. Please make yourself comfortable and don’t feel anxious. I will explain the exercise; please ask me if you have any questions about what you are supposed to do. At this station we will simulate the neonatal resuscitation. We will work with mannequins, but when you do the exercise, please act as if you were working with an actual patient. Imagine that you are in a health facility in a rural area with only basic equipment and that the mother is present.**

The equipment available to you is: a rubber bulb syringe, infant face mask, and Ambu bag, but no oxygen. Remember that the mother is present.

**Once again, we assume that you have taken the necessary infection prevention measures and that you have gathered the necessary equipment and supplies. Just like in the previous exercise, please demonstrate the steps in what you feel is the correct order. As you go along, explain the details of everything you are doing, even if it seems insignificant or routine. If you need help with anything, you can pretend that I am your assistant and tell me what you need me to do. Once you have finished, please tell me what additional steps you would take or assign to another person.**

**Do you have any questions?**

*Observer: answer any questions the participant might have. Once the participant has no more questions to ask say:*

**You may begin whenever you are ready.**

*Keep the mannequin out of the participant’s sight until he/she says that he/she is ready to begin. Then hand the mannequin to the participant just as if you were handing a newly delivered baby to an assistant after doing a delivery.*

| **Resuscitation using Ambu** | **EVALUATION** | | | |
| --- | --- | --- | --- | --- |
|  | **Done correctly** | **Done Incorrectly** | **Not done** | **Not observed** |
| 1. Places the newborn’s head in slightly extended position so as to open the airway. |  |  |  |  |
| 1. Covers the newborn, except the face and chest. |  |  |  |  |
| 1. Permeates the air passage with suction, first from the mouth and then from the nose. |  |  |  |  |
| 1. Reevaluates the newborn after the suction and gentle tactile stimulation. |  |  |  |  |
| 1. If the newborn is not breathing, he/she places the mask over the baby’s face so that it covers the chin, mouth, and nose. |  |  |  |  |
| 1. Establishes a seal between the mask and the baby’s face. |  |  |  |  |
| 1. Verifies the seal by ventilating with 2 fingers (or the entire hand, depending on the size of the bag) compressing the bag 2 or 3 times and watching for the chest to rise. |  |  |  |  |
| 1. If the newborn’s chest does not rise, he/she repositions the head, suctions and tries again to ventilate. |  |  |  |  |
| 1. Once the seal has been established, he/she ventilates (squeezing the bag), approximately 40 to 60 times per minute (short, rapid and delicately). |  |  |  |  |
| 1. After 30 seconds, he/she evaluates by watching the respiration, heart rate, and coloring. |  |  |  |  |

***Observer: Ask the participant:*** “How would you know or recognize if the resuscitation was successful, and what would you do in this case?”

|  | **Done correctly** | **Done Incorrectly** | **Not done** | **Not observed** |
| --- | --- | --- | --- | --- |
| 1. The breathing would be stable (> 30 per minute). |  |  |  |  |
| 1. Heart rate would be more than 100 per minute. |  |  |  |  |
| 1. The newborn’s coloring would be pink. |  |  |  |  |
| 1. Place the baby in direct contact (skin against skin) with the mother. |  |  |  |  |
| 1. Closely monitor newborn’s respiration for 5 minutes. |  |  |  |  |

*Observer: Ask the participant:* **“How would you know or recognize if the resuscitation was not successful, and what would you do in this case?”**

|  | **Done correctly** | **Done Incorrectly** | **Not done** | **Not observed** |
| --- | --- | --- | --- | --- |
| 1. A rapid assessment shows that the baby is not breathing, or the rate of respiration is less than 30 per minute. |  |  |  |  |
| 1. The heart rate is less than 100 per minute. |  |  |  |  |
| 1. The newborn’s coloring is bluish or pale. |  |  |  |  |
| 1. I would continue with the ventilation at 40 to 60 compressions per minute. |  |  |  |  |
| 1. Make the necessary preparations for the transfer of the newborn to a referral facility. |  |  |  |  |

# Continue on next page

# *Observer: Ask the participant:* “What more would you do when the procedure is finished? Remember to tell me when you have finished the procedure.”

|  | **EVALUATION** | |
| --- | --- | --- |
|  | **YES** | **NO** |
| 21. Inform the mother about what has happened |  |  |
| 22. Write information relevant to the mother and newborn in the clinical record. |  |  |
| 23. Make sure that the equipment is appropriately decontaminated and clean. |  |  |

**“Thank you very much for your participation, Dr./ Mr./Ms. _____. You have now completed all the skills evaluations and are free to go. Please do not discuss what you have done with anyone who is still waiting. You may discuss freely with anyone like you who has completed all the exercises.**

**Comments about the participant’s performance:**
