# Supplementary material for: Observe Before You Leap: Why Observation Provides Critical Insights for Formative Research and Intervention Design That You'll Never Get From Focus Groups, Interviews, or KAP Surveys
Source: Glob Health Sci Pract. 2018 Jun 27;6(2):299–316. doi: 10.9745/GHSP-D-17-00328 (PMC6024634; doi:10.9745/GHSP-D-17-00328)
Supplement: 17-00328-Harvey-Supplement1.docx [file 17-00328-Harvey-Supplement1.docx]

| 1. Number of Birds Observed by Species and Age | | | | | | | |
| --- | --- | --- | --- | --- | --- | --- | --- |
| Type of Bird | # Total | # Adult Males | # Adult Females | # Adult females sitting on eggs | # Immature Birds | # recently hatched chicks | # Corrals Observed |
| Chickens |  |  |  |  |  |  |  |
| Ducks |  |  |  |  |  |  |  |
| Turkeys |  |  |  |  |  |  |  |
| Other: |  |  |  |  |  |  |  |
| Other: |  |  |  |  |  |  |  |

| 2. When the interviewer arrived, were other animals present? | |
| --- | --- |
| Type of Animal | How many were there? |
| a. Dogs |  |
| b. Cats |  |
| c. Pigs |  |
| d. Guinea Pigs |  |
| e. Pigeons |  |
| f. Other (describe) |  |
| g. Other (describe) |  |

| 3. When the interviewer arrived, where were the birds?  For each corral, use the following codes:  (1) Corralled   (2) Free outside the house  (3) Free inside the house   (4) Some corralled and some free outside the house   (5) Some corralled and some free inside the house | Corral # | Code 1-4 |
| --- | --- | --- |
|  | 1 |  |
|  | 2 |  |
|  | 3 |  |
|  | 4 |  |
|  | 5 |  |

| 4. When the interviewer arrived, was there evidence that the birds were inside the house? (ex. Feces or feathers on the house floor)   *Mark the appropriate response with an X.*  ___ Yes (1) ___ No (2) |
| --- |

| 5. When the interviewer arrived, were the children around the house?   *Mark the appropriate response with an X.*  ___ Yes (1) ___ No (2) |
| --- |

| 6. When the interviewer arrived, how did the children interact with the birds?   *Mark* ***all of the appropriate responses*** *with an X.* |
| --- |
| ___ a. Children are in contact with the corralled birds.  ___ b. Children are in contact with the birds in the patio (outside the corral and outside the house)  ___ c. Children are in contact with the birds outside the corral and inside the house  ___ d. Children are not in contact with the birds, but are in contact with bird feces  ___ e. No contact is observed between the children and birds |

| 7. Which ages of children have contact with birds? (*Mark* ***all of the appropriate options*** *with an X.)* |
| --- |
| ____ a. Younger than 2 years ____ c. Older than 5 years  ____ b. Older than 2 years but younger than 5 years ____ d. No contact observed between children & birds |

| 8. Condition of the patio around the corral. (*Mark the appropriate column with an X. )* | | | | |
| --- | --- | --- | --- | --- |
|  | None (1) | Small amt (2) | Medium amt (3) | Large amt (4) |
| 1. Feces on the floor |  |  |  |  |
| 1. Food on the floor   *(vegetables, fruit peels, etc.)* |  |  |  |  |
| 1. Wet floor |  |  |  |  |
| 1. Flies/flees/other insects |  |  |  |  |
| 1. Ammonia odor (of feces) |  |  |  |  |
| 1. Other materials on the floor |  |  |  |  |

| 9. Is the structure of the corral in a good state of repair? | | |
| --- | --- | --- |
|  | Yes (1) | No (2) |
| a. Roof |  |  |
| b. Frame |  |  |
| c. Door |  |  |
| d. Front wall |  |  |
| e. Back wall |  |  |
| f. Left side wall |  |  |
| g. Right side wall |  |  |
| h. Other (describe): |  |  |
|  |  |  |
|  |  |  |

| 10. Condition inside the corral. (*Mark the appropriate column with an X.)* | | | | |
| --- | --- | --- | --- | --- |
|  | None (1) | Small amt (2) | Medium amt (3) | Large amt (4) |
| 1. Feces on the floor |  |  |  |  |
| b. Food on the floor  *(vegetables, fruit peels, etc.)* |  |  |  |  |
| c. Wet floor |  |  |  |  |
| d. Flies/flees/other insects |  |  |  |  |
| e. Ammonia odor (of feces) |  |  |  |  |
| f. Presence of shavings or other ground cover |  |  |  |  |
| g. Other material on the floor |  |  |  |  |

| 11. Food dish:   *Mark the appropriate box with an X.* | | | | |
| --- | --- | --- | --- | --- |
| a. Location | | | | |
| Inside the corral | | Outside the corral | | No food dish |
| Attached (1) | Loose (2) | Attached (3) | Loose (4) | (5) |
|  |  |  |  |  |
| b. Is it clean? | | Yes (1) | No (2) |  |
| c. Is there food in the food bowl? | | Yes (1) | No (2) |  |

| 12. Water dish:   *Mark the appropriate box with an X.* | | | | |
| --- | --- | --- | --- | --- |
| a. Location | | | | |
| Inside the corral | | Outside the corral | | No water dish |
| Attached (1) | Loose (2) | Attached (3) | Loose (4) | (5) |
|  |  |  |  |  |
| b. Is it clean? | | Yes (1) | No (2) |  |
| c. Is there water in the water bowl? | | Yes (1) | No (2) |  |

| 13. Condition of the Water  *Mark the appropriate box with an X.* | | | | |
| --- | --- | --- | --- | --- |
|  | None (1) | Small amt (2) | Medium amt (3) | Large amt (4) |
| a. Food in the water |  |  |  |  |
| b. Feces in the water |  |  |  |  |
| c. Dirt in the water |  |  |  |  |
| d. Other material in the water |  |  |  |  |

| 14. Is there a nest/area/structure for newly hatched birds?  *Mark the appropriate box with an X.*  ___ Yes (1) ___ No (2) |
| --- |

***Note: If so, continue with the questions below. If not, the observation ends here.***

| 15. Where is the area for newborn birds located?  ___ Inside the corral (1)  ___ Outside the corral, but inside the house (2)  ___ Outside the corral and outside the house (3) |
| --- |

| 16. What material(s) is it made out of?  *Mark the appropriate box with an X.*  ___ Cardboard (1) ____ Wood (2) ___ Cardboard Box (3)   ___ Fruit Box (4) ____ Other (5) |
| --- |

| 17. What is used to insulate it?  *Mark the appropriate box with an X.*  ___ Rag (1) ___ Rope (2) ___ Newspaper (3) ___ Sweater (4)  ___ Sawdust (5) ___ Shavings (6) ___ Other (7) |
| --- |

| 18. Is it deep enough that the birds cannot escape?  ___ Yes (1) ___ No (2) ___ Not possible to determine (3) |
| --- |

| 19. Conditions inside the nest/structure for the newborn birds | | | | |
| --- | --- | --- | --- | --- |
|  | None (1) | Small amt (2) | Medium amt (3) | Large amt (4) |
| a. Feces on the floor |  |  |  |  |
| b. Food on the floor |  |  |  |  |
| c. Wet floor |  |  |  |  |
| d. Flies/fleas/other insects |  |  |  |  |
| e. Ammonia odor (of feces) |  |  |  |  |
| f. Presence of wood shavings or other cover |  |  |  |  |
| g. Other materials on the floor (describe) |  |  |  |  |
