# Supplementary material for: Observe Before You Leap: Why Observation Provides Critical Insights for Formative Research and Intervention Design That You'll Never Get From Focus Groups, Interviews, or KAP Surveys
Source: Glob Health Sci Pract. 2018 Jun 27;6(2):299–316. doi: 10.9745/GHSP-D-17-00328 (PMC6024634; doi:10.9745/GHSP-D-17-00328)
Supplement: 17-00328-Harvey-Supplement2.docx [file 17-00328-Harvey-Supplement2.docx]

**Direct observation of bed net use in the target communities**

***All the information should be based on observation, not on participant report unless otherwise indicated.***

1. Type of bed

| Composition | Bed #1 | Bed #2 | Bed #3 | Bed #4 | Bed #5 |
| --- | --- | --- | --- | --- | --- |
| Wooden Floor (boards) |  |  |  |  |  |
| *Pona* Floor |  |  |  |  |  |
| Dirt Floor |  |  |  |  |  |
| Platform bed with wooden slats |  |  |  |  |  |
| Platform bed with *pona* slats |  |  |  |  |  |
| Platform bed with foam mattress |  |  |  |  |  |
| Platform bed with some other type of mattress |  |  |  |  |  |
| Other type of bed (describe) |  |  |  |  |  |

1. Are there holes in the bed? (ex. Between the slats or *pona* strips)

|  | Bed #1 | Bed #2 | Bed #3 | Bed #4 | Bed #5 |
| --- | --- | --- | --- | --- | --- |
| Yes = 1; No = 2 |  |  |  |  |  |

1. Is a sheet used on the bed?

|  | Bed #1 | Bed #2 | Bed #3 | Bed #4 | Bed #5 |
| --- | --- | --- | --- | --- | --- |
| Yes = 1; No = 2 |  |  |  |  |  |

1. According to the ***female head of household (report),*** who sleeps in each bed?

|  | Bed #1 | Bed #2 | Bed #3 | Bed #4 | Bed #5 |
| --- | --- | --- | --- | --- | --- |
| Record relation and age (ex.: mother, son 2 years, daughter 1 year) |  |  |  |  |  |

1. According to ***direct observation***, who sleeps in each bed?

|  | Bed #1 | Bed #2 | Bed #3 | Bed #4 | Bed #5 |
| --- | --- | --- | --- | --- | --- |
| Record relation and age (ex.: mother, son 2 years, daughter 1 year) |  |  |  |  |  |

1. What type of bed net is used on each bed?

|  | Bed #1 | Bed #2 | Bed #3 | Bed #4 | Bed #5 |
| --- | --- | --- | --- | --- | --- |
| Single-ply muslin fabric |  |  |  |  |  |
| Double-ply muslin fabric |  |  |  |  |  |
| Nylon |  |  |  |  |  |
| Other (describe below) |  |  |  |  |  |
| None |  |  |  |  |  |

Description:

1. ***According direct observation,*** at what time is the bed net lowered over the bed?

|  | Bed #1 | Bed #2 | Bed #3 | Bed #4 | Bed #5 |
| --- | --- | --- | --- | --- | --- |
| Time (hh:mm) (if all the people in bed do not go to bed at the same time, record the time each person goes to bed) |  |  |  |  |  |

1. How are the borders of the bed net secured to the bed?

|  | Bed #1 | Bed #2 | Bed #3 | Bed #4 | Bed #5 |
| --- | --- | --- | --- | --- | --- |
| Describe the way the borders are secured or if they are not, record that. |  |  |  |  |  |

1. At what time did each family member go to bed?

| Father | Mother | Child #1 | Child #2 | Child #3 | Child #4 | Child #5 | Child #6 |
| --- | --- | --- | --- | --- | --- | --- | --- |
|  |  |  |  |  |  |  |  |

1. Record the activity of each household member from 5:30 p.m. until everyone has gone to bed. Note each change in activity, reporting the time it occurs adding a note at least every 5 minutes. Especially pay attention to each activity that could affect the contact between human and mosquito (ex. Person exiting the bed net after they already went to bed; person leaving the bed during the night; returning after 5:30pm or leaving early in the morning.

| Time (hh:mm) | Activity |
| --- | --- |
|  |  |
